# Bta-miR-24-3p Controls the Myogenic Differentiation and Proliferation of Fetal Bovine Skeletal Muscle-Derived Progenitor Cells by Targeting *ACVR1B*

**DOI:** 10.3390/ani9110859

**Published:** 2019-10-24

**Authors:** Xin Hu, Yishen Xing, Ling Ren, Yahui Wang, Qian Li, Xing Fu, Qiyuan Yang, Lingyang Xu, Luc Willems, Junya Li, Lupei Zhang

**Affiliations:** 1 Institute of Animal Sciences, Chinese Academy of Agricultural Sciences, Beijing 100193, China; huxin19890803@163.com (X.H.); yishen_xing@163.com (Y.X.); renling5454@163.com (L.R.); wang1434243198@163.com (Y.W.); lq798711247@163.com (Q.L.); xulingyang@caas.cn (L.X.); 2 Molecular and Cellular Biology, Gembloux Agro-Bio Tech, University of Liège, 5030 Gembloux, Belgium; luc.willems@uliege.be; 3 School of Animal Sciences, Louisiana State University Agricultural Center, 229G Animal and Food Sciences laboratory building, Baton Rouge, LA 70803, USA; XFu1@agcenter.lsu.edu; 4 Department of Molecular, Cell and Cancer Biology, University of Massachusetts Medical School, Worcester, MA 01605, USA; Qiyuan.Yang@umassmed.edu

**Keywords:** bta-miR-24-3p, bovine, fetal skeletal muscle, proliferation, differentiation, *ACVR1B*

## Abstract

**Simple Summary:**

MicroRNAs play pivotal roles in skeletal muscle development, but the molecular basis of their functions in fetal bovine skeletal muscle development is largely unknown. Here, we report a mechanistic study of bta-miR-24-3p, a key miRNA regulator of the myogenic differentiation of fetal bovine platelet-derived growth factor receptor alpha negative (PDGFRα^-^) progenitor cells. We isolated progenitor cells from the bovine fetal longissimus dorsi muscle and purified them with PDGFRα antibodies to remove fibro-adipogenic progenitors. We observed elevated bta-miR-24-3p expression during differentiation, and bta-miR-24-3p overexpression led to promoted myogenic differentiation but suppressed proliferation. Moreover, activin receptor type 1B (*ACVR1B*) was identified as a direct target of bta-miR-24-3p, and *ACVR1B*-silencing cells exhibited similar phenotypes to bta-miR-24-3p-overexpressing bovine PDGFRα^-^ progenitor cells. These results extended our understanding on the roles of miRNA in fetal muscle development. The method of removing fibro-adipogenic progenitors in our study will also provide useful information for other investigators.

**Abstract:**

MicroRNAs modulate a variety of cellular events, including skeletal muscle development, but the molecular basis of their functions in fetal bovine skeletal muscle development is poorly understood. In this study, we report that bta-miR-24-3p promotes the myogenic differentiation of fetal bovine PDGFRα^-^ progenitor cells. The expression of bta-miR-24-3p increased during myogenic differentiation. Overexpression of bta-miR-24-3p significantly promoted myogenic differentiation, but inhibited proliferation. A dual-luciferase assay identified *ACVR1B* as a direct target of bta-miR-24-3p. Similarly, knocking down *ACVR1B* by RNA interference also significantly inhibited proliferation and promoted the differentiation of bovine PDGFRα^-^ progenitor cells. Thus, our study provides a mechanism in which bta-miR-24-3p regulates myogenesis by inhibiting *ACVR1B* expression.

## 1. Introduction

The fetal stage is crucial for skeletal muscle development, because the majority of skeletal muscle fibers form during this stage [1]. Skeletal muscle growth and development involve a series of complex biological processes regulated by myogenic regulatory factors (MRFs) [2]. For example, myogenic factor 5 (*Myf5*) and myogenic differentiation 1 (*MyoD*) are critical for the commitment, proliferation, and survival of myoblasts, whereas myogenin (*MyoG)* and myogenic factor 6 (*Myf6*) play essential roles in terminal differentiation [3,4,5]. Myogenic differentiation is also regulated at the post-transcriptional level, such as with microRNAs (miRNAs).

MicroRNAs (miRNAs) are endogenous, non-coding RNAs (ncRNA) of 20–24 nucleotides, and suppress translation via binding to the 3′-untranslated region (3′-UTR) of their target mRNAs [6,7]. It has been reported that miRNAs regulate muscle development [8,9]. For example, miR-143 regulates bovine muscle satellite cell (MSCs) differentiation and proliferation by directly targeting insulin like growth factor binding protein 5 (*IGFBP5)* [10]. miR-148-3p could inhibit the proliferation and enhance the apoptosis of bovine muscle cells by targeting Kruppel like factor 6 (*KLF6*) [11]. bta-miR-378 could promote bovine skeletal muscle-derived satellite cell (bMDSC) differentiation by suppressing DNA polymerase alpha 2 (*POLA2*) expression [12]. These findings point to a role of miRNAs in bovine skeletal muscle development.

bta-miR-24-3p is 22 nt in length and is derived from its precursor miRNA bta-miR-24-2. miR-24 is upregulated during myogenesis and promotes myogenic differentiation through an unknown mechanism [13]. According to published data, miR-24-3p hampers skeletal muscle fibrosis by interacting with its target, *Smad2*, which is involved in transforming growth factor-β (TGFβ)/Smad signaling [14]. However, the regulatory role of bta-miR-24-3p in the prenatal development of bovine skeletal muscle is still not clear. 

Activin receptor type 1B (*ACVR1B*, also known as *ALK4*) encodes a transducer of activin or activin-like ligand signals. It is known to participate in embryonic development, nervous system differentiation, germ cell development, tumor formation, and immunosuppression [15,16,17]. *ACVR1B* influences myogenesis and regulates the balance between protein synthesis and degradation by modulating the *MSTN* pathway to maintain muscle mass [18]. In addition, miR-210 regulates transforming growth factor-β (TGF-β)/activin signaling to promote osteoblastic differentiation by targeting *ACVR1B* [19]. A study with *ACVR1B*-knockout mice provided evidence for a specific and indispensable role of *ACVR1B* signaling in the cycling and differentiation of hair follicle and tooth morphogenesis [20]. However, the role of *ACVR1B* in fetal bovine myogenesis and proliferation, and whether it is regulated by miRNAs in the differentiation and proliferation of skeletal muscle, is still unknown.

In this study, we purified myogenic progenitor cells using antibodies of platelet-derived growth factor receptor alpha (PDGFRα), which is the cell surface marker of fibro/adipogenic lineages [21], and named the cells as PDGFRα^-^ progenitor cells. This study investigates the underlying molecular basis of how miR-24-3p modulates the differentiation and proliferation of fetal bovine skeletal, muscle-derived progenitor cells. Moreover, we predicted the potential targets of bta-miR-24-3p and experimentally demonstrated its regulatory mechanism. The effect of *ACVR1B* on the differentiation and proliferation of fetal bovine skeletal muscle-derived progenitor cells was also explored. Our results demonstrate that bta-miR-24-3p inhibits bovine PDGFRα^-^ progenitor cell proliferation and improves their differentiation by targeting *ACVR1B*. This research provides insight into the epigenetic regulation of skeletal muscle development during the fetal stage.

## 2. Materials and Methods

### 2.1. Ethics Statement

All animal experiments followed the guidelines of the Regulations for the Administration of Affairs Concerning Experimental Animals (Ministry of Science and Technology, China, 2004). All animal experimental protocols in this study were strictly monitored by the Animal Ethics Committee of the Institute of Animal Sciences, Chinese Academy of Agricultural Sciences (IAS-CAAS). Ethical approval of animal survival was given by the animal ethics committee of IAS-CAAS (No. IAS2019-48). Pregnant cows were raised at the Inner Mongolia Aokesi Agriculture Co., Ltd. (Wulagai, China). Efforts were made to minimize the cows’ suffering.

### 2.2. Primary Cell Isolation and Cell Culture

The progenitor cells were enzymatically isolated from longissimus dorsi tissues obtained from three male bovine fetuses about 90–120 days after conception, as described previously [22]. Briefly, longissimus dorsi was minced into small fragments and then incubated with 0.1% type IV collagenase (Sigma, St. Louis, MO, USA) for 1 h. This enzymatic digestion was terminated by adding cell culture medium containing low-glucose DMEM (Dulbecco’s Modified Eagle medium; Gibco, Grand Island, NY, USA), supplemented with 10% FBS (fetal bovine serum; Gibco, Grand Island, NY, USA). The digested tissue suspension was passed through a nylon mesh (40 μm pore size), and the pellets were obtained by centrifuging and resuspending in ice-cold phosphate-buffered saline (PBS) buffer containing 2 mM EDTA and 0.5% bovine serum albumin. The cell suspension was incubated with anti-PDGFRα antibody (Cell Signaling Technology, Danvers, MA, USA) at 4 °C for 30 min. After washing and resuspending in PBS, the cells were incubated with Anti-Rabbit IgG MicroBeads (Miltenyi Biotec, Bergisch Gladbach, Germany) at 4 °C for 15 min. Subsequently, the cells were harvested and resuspended in the buffer. PDGFRα- cells were separated by a MACS column (Miltenyi Biotec) and magnetic MiniMACS Separator (Miltenyi Biotec). These cells were cultured in DMEM supplemented with 10% FBS and maintained at 37 °C with a 5% CO_2_ atmosphere. The cells from each fetus were maintained separately. The cells were passaged with 0.25% trypsin-EDTA (Gibco, Grand Island, NY, USA) at 70–80% confluence and induced in the differentiation medium (DM) containing Dulbecco’s modified Eagle’s medium (DMEM, Gibco, Grand Island, NY, USA) and 5% horse serum when reaching 100% confluence.

DMEM was used to culture HEK293, a human embryonic kidney cell line. The medium contains high glucose, 100 U/mL penicillin, 10% fetal bovine serum (FBS, Gibco, Grand Island, NY, USA), and 100 μg/mL streptomycin at 37 °C and 5% CO_2_.

### 2.3. RNA Isolation, Reverse Transcription (RT), and Quantitative Real-Time PCR (qRT-PCR)

Total RNA was isolated from the cells using the TRIzol reagent (Invitrogen, Carlsbad, CA, USA). RNA concentration and quality were assessed by a NanoPhotometer N50 (Implen, Munich, Germany) and 1.5% agarose gel electrophoresis. We synthesized first-strand cDNA by reverse-transcribing the isolated total RNA using PrimeScript RT Master Mix (Perfect Real Time) (TaKaRa, Kusatsu, Japan). For MicroRNA, the reverse transcripts were obtained with a miRcute Plus miRNA First-Stand cDNA Synthesis Kit (KR211, TIANGEN, Beijing, China). Then quantitative real-time PCR (qRT-PCR) was performed by the QuantStudio 7 Flex Real-Time PCR System (Life, Carlsbad, CA, USA), with the KAPA SYBR^®^ FAST qPCR Kit (KAPABiosystems, Wilmington, MA, USA) and a miRcute Plus miRNA qPCR Kit (FP411, TIANGEN). All assays were performed following the manufacturer’s recommendations. Three technical replicates were used for all reactions. The sequences of miRNA-specific and qRT-PCR primers are summarized in Table 1.

### 2.4. Immunofluorescence

For immunofluorescence, the cells were cultured in 12-well plates, fixed in 4% paraformaldehyde for 15 min, and washed three times (five min each) with PBS. Next, we permeabilized the cells with 0.1% Triton X-100 for 10 min and blocked the cells with 1% albumin bovine serum (Beyotime, Shanghai, China) for 30 min. After incubation with the anti-myosin heavy chain (MHC) antibody (1:100, Developmental Studies Hybridoma Bank, Iowa, USA) overnight at 4 °C, FITC-labeled Goat Anti-Mouse IgG (H + L) (1:1000, Beyotime) was added, and the cells were incubated at room temperature for 1 h. The cell nuclei were stained with DAPI (Sigma-Aldrich, St. Louis, MO USA) for 5 min, and images were obtained with a confocal microscope (TCS SP8, Leica, Wetzlar, Germany).

### 2.5. Western Blot 

The cells were lysed with proteinase inhibitors, and protein samples of equal amount were separated on 4–12% SurePAGE gels (GenScript, Nanjing, China). The proteins were transferred to nitrocellulose membranes (Pall, Mexico) after electrophoresis and blocked in 5% (*w*/*v*) non-fat milk. The membranes were incubated with the ACVR1B (1:3000, Proteintech, Chicago, IL, USA), MyoG (1:1000, Santa Cruz, Delaware Ave Santa Cruz, CA, USA), MHC (1:50, Developmental Studies Hybridoma Bank), and β-tubulin (1:2000, Proteintech) antibodies—all of which are primary antibodies—at 4 °C overnight, washed three times with saline/Tween (in Tris), and incubated with HRP-labeled Goat Anti-Mouse IgG (1:1000, Beyotime) or HRP-labeled Goat Anti-Rabbit IgG (1:1000, Beyotime) at room temperature for 1 h. ECL western blotting detection reagent (Beyotime) was used to visualize the protein bands.

### 2.6. 5-Ethynyl-2′-deoxyuridine (EdU) Assay

The cells were cultured until 50% density and transfected with the miR-24-3p mimic, the mimic NC, the ACVRIB siRNA (si-*ACVR1B*), and the siRNA negative control (si-NC). The cells were subjected to the 5-Ethynyl-2’-deoxyuridine (EdU) assay 24 h after transfection using an EdU Apollo In Vitro Imaging Kit (RiboBio, Guangzhou, China). We captured three randomly selected fields using a fluorescence microscope (TCS SP8, Leica) and determined the number of EdU-stained cells. ImageJ was used to determine the percentage of EdU-positive cells.

### 2.7. Cell Counting Kit-8 (CCK8) Assay

The cells were cultured in 96-well plates and transfected with the miR-24-3p mimic or the mimic NC. After 24, 48, 72, and 96 h of transfection, a 10 μL Cell Counting Kit-8 reagent (Beyotime) was added into each well and incubated for 1 h. Optical absorption at 450 nm (A450) was measured by an Imark Microplate Reader (Bio-Rad), and the average A450 values of six independent experiments were calculated.

### 2.8. RNA Oligonucleotide Preparation and Cell Transfection

The bta-miR-24-3p mimics, the mimic NC, the siRNAs used to knockdown *ACVR1B*, and the non-specific siRNA negative control were designed and synthesized by RiboBio (Guangzhou, China). The si-*ACVR1B* sequence is 5’-CGCTGACAATAAAGATAAC-3’. Transfection was performed with the Lipofectamine RNAiMAX reagent (Invitrogen). All procedures were performed according to the manufacturers’ protocols.

### 2.9. Prediction of miRNA Target Genes

The miRNA target gene prediction was performed by TargetScanHuman 7.2 (http://www.targetscan.org/vert_72/). 

### 2.10. Dual-Luciferase Reporter Assay

The binding site of bta-miR-24-3p in *ACVR1B* was amplified from bovine DNA and inserted into the psi-CHECK2 vector (Promega, Madison, WI, USA) via XhoI and NotI double digestion. Site-directed mutagenesis of the resulting construct was performed using the Fast Site-Directed Mutagenesis Kit (TIANGEN) to remove the potential binding site. Refer to Table 2 for details on primers used in plasmid construction and mutagenesis.

For luciferase reporter assays, HEK293T cells were co-transfected with bta-miR-24-3p mimics (or the mimic NC) and the wild-type or mutant psi-CHECK2 plasmid, using the Lipofectamine 3000 reagent (Invitrogen). Luciferase activity was determined by the Dual-Luciferase Reporter Assay System (Promega) 48 h after transfection.

### 2.11. Statistical Analysis 

At least three technical replicates were used for statistical analysis. The Normality of the data was verified by Student’s *t*-test using GraphPad Prism (version 6.0, GraphPad Software, San Diego, CA, USA); *p* < 0.05 was considered statistically significant.

## 3. Results

### 3.1. Bta-miR-24-3p Is Up-Regulated During the Myogenic Differentiation of PDGFRα^-^ Progenitor Cells

**Figure 1:**
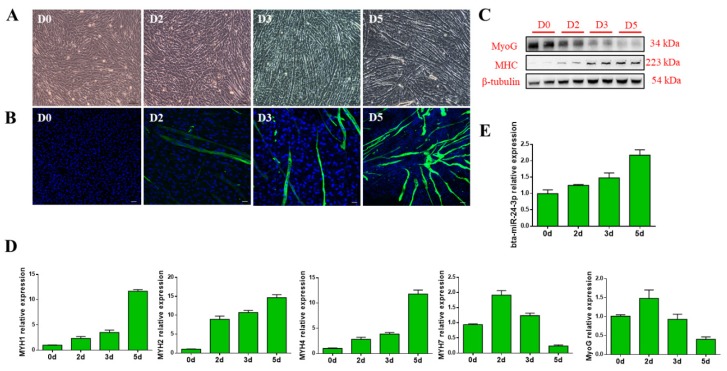
bta-miR-24-3p expression during the myogenic differentiation of platelet-derived growth factor receptor alpha (PDGFRα-) progenitor cells. (**A**) Microscopic images of bovine PDGFRα^-^ progenitor cells on days 0, 2, 3, and 5 (D0, D2, D3, and D5, respectively) of differentiation. Scale bars = 100 µm. (**B**) Myosin heavy chain (MHC)-positive cells (green) on D0, D2, D3, and D5 of myogenic differentiation, visualized by immunofluorescence; scale bars = 100 µm. (**C**) Western blot evaluating the protein levels of myogenin and MHCs in cells cultured, as described in A. (**D**) Transcript levels of myogenin and MHCs in cells cultured, as described in (A). (**E**) The transcript level of bta-miR-24-3p in cells cultured, as described in (A). All data are represented as mean ± standard deviation (SD), based on at least three independent experiments for each treatment.

To investigate the expression of bta-miR-24-3p during myogenesis, PDGFRα^-^ progenitor cells were isolated from the longissimus dorsi tissue of bovine fetus, according to a previous study [21], and then myogenic differentiation was induced in vitro. The PDGFRα^-^ progenitor cells formed obvious myotubes two days after myogenic induction (Figure 1A,B). Moreover, immunostaining of muscle-specific protein showed that MyoG was downregulated during myogenic differentiation, whereas myosin heavy chain (MHC) was upregulated (Figure 1C). We then determined the transcript levels of the *MYH* genes during myogenic differentiation, and found that the *MYH1*, *MYH2* and *MYH4* expression increased, whereas that of *MYH7* decreased two days after differentiation (Figure 1D). In addition, a gradual increase in bta-miR-24-3p expression was observed during myogenic differentiation (Figure 1E).

### 3.2. Bta-miR-24-3p Promotes the Myogenic Differentiation of Bovine PDGFRα^-^ Progenitor Cells

**Figure 2:**
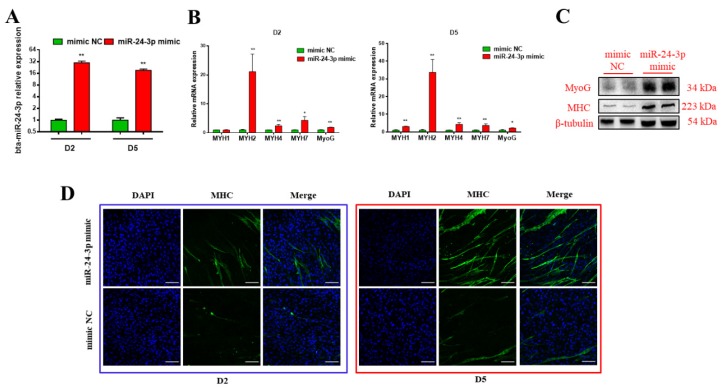
The expression of bta-miR-24-3p during the myogenic differentiation of bovine PDGFRα^-^ progenitor cells. (**A**) bta-miR-24-3p expression 48 h after miR-24-3p mimics transfection, determined by qRT-PCR. (**B**) Transcript levels of myogenin and MHCs detected by qRT-PCR on D2 and D5. (**C**) Western blot evaluating the protein levels of myogenin and MHC 96 h following differentiation. (**D**) MHC-positive cells (green) on D2 and D5 of myogenic differentiation, visualized by immunofluorescence. Scale bars represent 100 µm. All data are represented as mean ± standard deviation (SD,) based on at least three independent experiments for each treatment. * *p* < 0.05 and ** *p* < 0.01.

To investigate the potential roles of bta-miR-24-3p in bovine skeletal muscle myogenesis during the fetal period, we transfected bta-miR-24-3p mimics and the negative control (NC) into PDGFRα^-^ progenitor cells. The levels of mature bta-miR-24-2 in the mimic group on day 2 and day 5 were 30- and 19-fold higher than those in the NC group, respectively (Figure 2A). bta-miR-24-3p accumulation led to a significant increase in the transcript levels of myogenic differentiation marker genes, including *MyoG*, *MYH1*, *MYH2*, *MYH4*, and *MYH7* (Figure 2B). Consistent with the results of transcript analysis, significantly higher levels of MyoG and MHC proteins were observed in the mimic group than in the NC group (Figure 2C). The immunofluorescence assay showed that bta-miR-24-3p mimics significantly increased the total number of MHC-positive cells at the end of myogenic differentiation, as compared with the control group (Figure 2D). Taken together, these results point to a role of bta-miR-24-3p in promoting myogenic differentiation.

### 3.3. Bta-miR-24-3p Inhibits Bovine PDGFRα^-^ Progenitor Cell Proliferation

**Figure 3:**
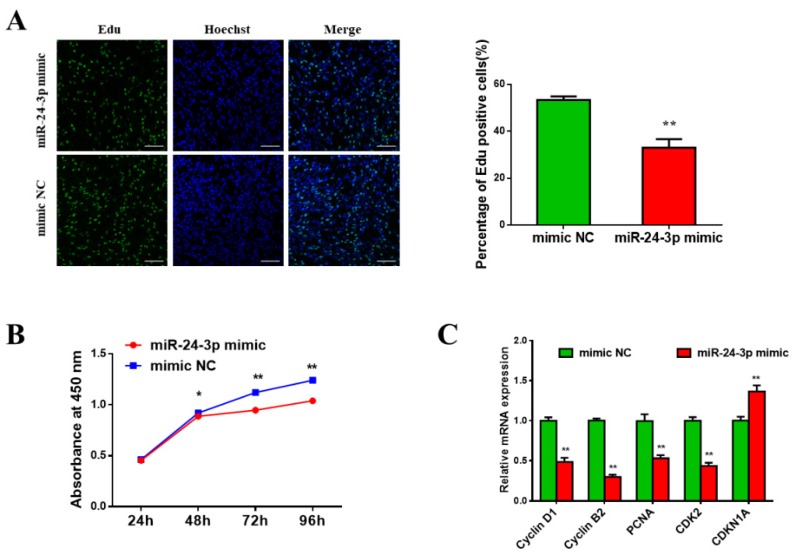
The effect of miR-24-3p on bovine PDGFRα^-^ progenitor cell proliferation. (**A**) EdU staining of the PDGFRα^-^ progenitor cells after the transfection of miR-24-3p mimics and EdU-positive cell determination. (**B**) Cell Counting Kit-8 (CCK8) assay showing that miR-24-3p mimics inhibit PDGFRα^-^ progenitor cell proliferation. (**C**) The relative transcript levels of cell cycle-related genes after transfection. Scale bars = 100 µm. All data are represented as mean ± standard deviation (SD), based on at least three independent experiments for each treatment. * *p* < 0.05 and ** *p* < 0.01.

To further investigate the role of bta-miR-24-3p in bovine PDGFRα^-^ progenitor cell proliferation, an EdU assay and CCK8 assay were used to analyze the proliferation of cells transfected by miR-24-3p mimics or NC. The EdU assays showed that miR-24-3p overexpression significantly decreased the proportion of EdU-positive cells (Figure 3A). The CCK8 assay revealed that miR-24-3p overexpression inhibited bovine PDGFRα- progenitor cell proliferation (Figure 3B). In addition, the transcript levels of cyclin D1, cyclin B1, proliferating cell nuclear antigen (*PCNA*), and cyclin-dependent kinase 2 (*CDK2*)—all of which are cell cycle-promoting genes—were decreased upon miR-24-3p overexpression. Conversely, miR-24-3p overexpression promotes the expression of the cell proliferation-inhibiting gene cyclin-dependent kinase inhibitor 1A (*CDKN1A*) (Figure 3C). These results demonstrate the inhibitory effect of bta-miR-24-3p on bovine PDGFRα^-^ progenitor cell proliferation.

### 3.4. ACVR1B Is a Direct Target of Bta-miR-24-3p

**Figure 4:**
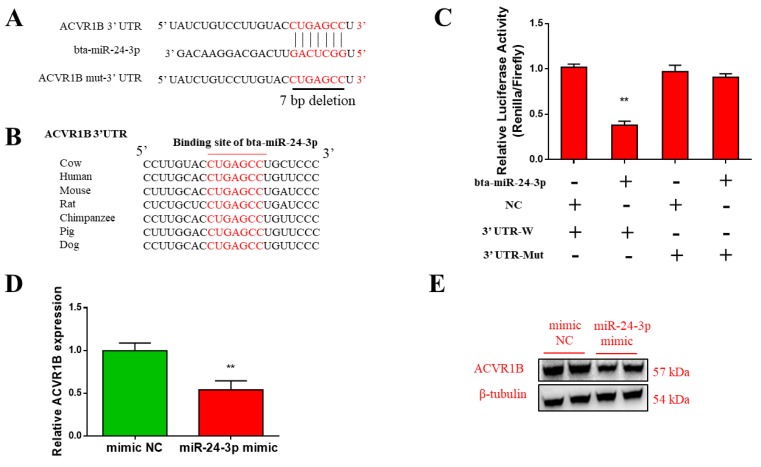
bta-miR-24-3p directly targets *ACVR1B* gene. (**A**) The predicted binding site of bta-miR-24-3p in *ACVR1B* 3’-UTR. The predict binding site (marked in red) was deleted in the mutated 3′-UTR reporter. (**B**) Sequence alignment of the mature bta-miR-24-3p binding sites in seven different species. (**C**) HEK293T cells were co-transfected with bta-miR-24-3p mimics, as well as the dual-luciferase reporters linked to the wild-type or mutant bta-miR-24-3p binding sites. Relative luciferase activity was analyzed 48 h after transfection (** *p* < 0.01). (**D** and **E**) The transcript and protein levels of ACVR1B in HEK293T cells transfected with bta-miR-24-3p mimics 48 h after transfection. All data are represented as mean ± standard deviation (SD), based on at least three independent experiments for each treatment. * *p* < 0.05 and ** *p* < 0.01.

To further explore the molecular mechanism of how bta-miR-24-3p regulates bovine myogenesis, we predicted miR-24-3p targets using the TargetScanHuman 7.2 databases (http://www.targetscan.org/vert_72/). The seed sequence of bta-miR-24-3p perfectly matches the 3′ untranslated region (UTR) of *ACVR1B*, suggesting that *ACVR1B* is a potential target of bta-miR-24-3p (Figure 4A). Furthermore, we found that the potential binding site of bta-miR-24-3p was highly conserved among mammalian *ACVR1B* genes (Figure 4B).

To verify the interaction between bta-miR-24-3p and *ACVR1B*, we inserted the fragment harboring either the wild-type or mutated bta-miR-24-3p binding sequence in *ACVR1B* into a dual-luciferase reporter vector. Each of the resulting constructs was separately introduced into 293T cells, along with bta-miR-24-3p mimics, and luciferase activity was detected 48 h after transfection. We detected significantly decreased luciferase activity in 293T cells expressing the wild-type *ACVR1B* 3’ UTR fragment, whereas that of 293T cells expressing the mutated fragment was not changed (Figure 4C). This result suggests that the analyzed 3’ UTR sequence of *ACVR1B* is involved in bta-miR-24-3p recognition. More importantly, compared with the control, we observed a significant reduction in the *ACVR1B* transcript level in bovine PDGFRα^-^ progenitor cells overexpressing bta-miR-24-3p (Figure 4D). The protein level of ACVR1B in PDGFRα^-^ progenitor cells was also decreased upon overexpressing miR-24-2 mimics (Figure 4E). Collectively, these results demonstrate that *ACVR1B* is a direct target of bta-miR-24-3p in bovine PDGFRα^-^ progenitor cells.

### 3.5. ACVR1B Silencing Promotes Myogenic Differentiation But Inhibits Proliferation

**Figure 5:**
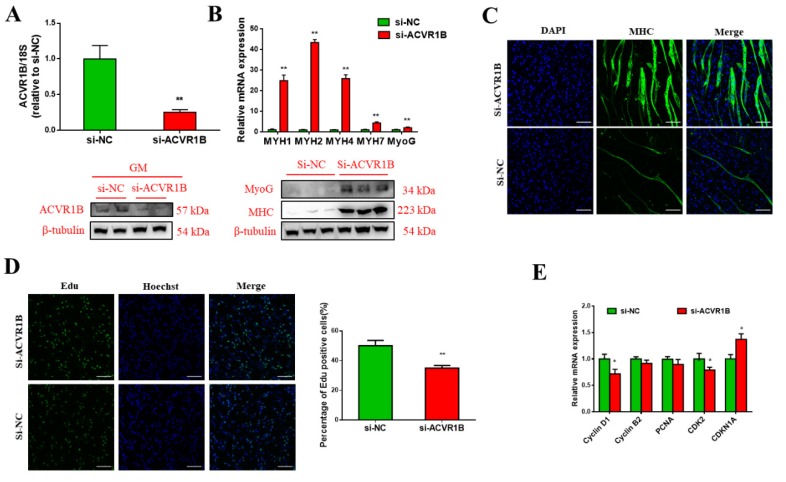
*ACVR1B* knockdown promotes myogenic differentiation, but inhibits proliferation. (**A**) The transcript and protein levels of ACVR1B in PDGFRα^-^ progenitor cells transfected with si-*ACVR1B* or si-NC. (**B**) The transcript and protein levels of MHC and MyoG on D5 of differentiation in bovine PDGFRα^-^ progenitor cells transfected with si-*ACVR1B* or si-NC. (**C**) MHC-positive cells on D5 of myogenic differentiation in PDGFRα^-^ progenitor cells transfected with si-*ACVR1B* and si-NC, as visualized by immunofluorescence. (**D**) EdU staining assay of PDGFRα^-^ progenitor cells transfected with si-*ACVR1B* or si-NC, and the determination of EdU-positive cells. (**E**) Relative transcript levels of cell cycle-related genes in PDGFRα^-^ progenitor cells transfected with si-*ACVR1B* or si-NC. Scale bars = 100 µm. All data are represented as mean ± standard deviation (SD), based on at least three independent experiments for each treatment. * *p* < 0.05 and ** *p* < 0.01.

To examine the role of *ACVR1B* in myogenic differentiation, we silenced the endogenous *ACVR1B* in bovine PDGFRα^-^ progenitor cells using small interfering RNA (siRNA). The interference efficiency was verified, and the siRNA against *ACVR1B* (si-*ACVR1B*) significantly decreased the transcript and protein levels of ACVR1B (Figure 5A). Moreover, a similar result was obtained on day 5 of myogenic differentiation, when si-*ACVR1B* significantly upregulated the transcript levels of myogenesis marker genes, *MyoG*, *MYH1*, *MYH2*, *MYH4*, and *MYH7* (Figure 5B). Consistently, the protein levels of MyoG and MHC in the myogenic differentiation increased upon *ACVR1B* knockdown (Figure 5B). Furthermore, more multinuclear myotubes were observed in the knockdown group (Figure 5C). The EdU assay revealed decreased number of EdU-positive cells in the si-*ACVR1B* group compared with the control (Figure 5D), along with reduced Cyclin D1 and *CDK2* but increased *CDKN1A* mRNA levels (Figure 5E). Together, these findings suggest that knocking down *ACVR1B* significantly promotes myogenic differentiation, but represses the proliferation of bovine PDGFRα^-^ progenitor cells.

## 4. Discussion

miR-24-2 is transcribed and processed from miR-23a/27a/24-2 clusters. It is suggested that miR-24-2 is involved in many physiological and pathological processes, including cell differentiation, proliferation, apoptosis, and metabolism, as well as disease occurrence [23,24,25,26]. miR-24-3p suppresses apoptosis by targeting the Keap1-Nrf2 Pathway [27]. In lung cancer cells, miR-24-3p could promote cell proliferation and migration; it also accelerates tumor growth in xenograft mice by directly binding to SRY-box transcription factor 7 *(SOX7)* [28]. MiR-24 also participates in erythropoiesis [29]. miR-24-3p controls osteogenic differentiation via regulating SMAD family member 5 (Smad5) in human periodontal ligament stem cells (hPDLSCs) [30]. Previous studies have demonstrated that miR-24-3p can promote the proliferation of several types of cancer cells [25,31,32,33,34,35], which contradicts our results. However, some studies have demonstrated that miR-24 could target high mobility group box 1 (*HMGB1*) and inhibit the proliferation and migration of vascular smooth muscle cells [36]. These conflicting results may be mainly due to the differences in the cell types used. Also, miR-24 was found to inhibit proliferation by prohibiting or delaying the G1-to-S transition [37]. Although the effect of miR-24-3p on proliferation and myogenic differentiation has been previously reported, its target remains unclear. Here, we identified *ACVR1B* as a direct target of bta-miR-24-3p using an in vitro model of fetal bovine myogenesis, and illustrated that bta-miR-24-3p inhibits proliferation and promotes myogenic differentiation of progenitor cells by targeting *ACVR1B*.

*ACVR1B* is a member of the TGF-β pathway, which transduces extracellular signaling into the cytoplasm [38]. TGF-β inhibits myogenesis through inhibiting the expression and function of MRFs. It has been shown that TGF-β reduces the expression of myogenin and myocyte enhancer factor 2D (MEF2D), as well as myotube formation in the C2C12 model via the Smad pathway, in a cell density-dependent manner [39]. In addition, TGF-β induces MEF2 translocation from the nucleus to the cytoplasm, thereby inhibiting MEF2 transcription [40]. It has also been demonstrated that TGF-β inhibits myogenic differentiation via intracellular effector Smad3 in C3H10T1/2 and C2C12 cell lines by repressing the activity of MyoD family members [41]. Moreover, TGF-β treatment inhibits the transcriptional upregulation of p21 and muscle creatinine kinase (MCK) in C2C12 cells, which are associated with growth arrest at the G1 phase and representative cell-lineage-specific expression during myogenesis [42]. Furthermore, TGF-β could induce miRNAs, such as miR-206 and miR-29, to control myogenic differentiation by regulating the expression of HDAC4 [43].

*ACVR1B* regulates the pluripotency and differentiation of stem cells in osteoclastogenesis, myogenesis, and adipogenesis [44,45,46]. The activated type I receptor phosphorylates and thereby activates specific Smads in the canonical signaling pathway, which enter into the nucleus to control the gene transcriptional cascades [38,47,48]. Although several types of ligands share common receptors or downstream Smads, they could fulfill their essential functions *in vivo* without interference from the many other proteins of the superfamily. The ligands structure evolution enables distinct modes of receptor binding, and engenders the TGF-βs with high specificity for their receptors [49]. The SMAD complexes have weak affinity for DNA and limited specificity, and need cooperate with other site-specific transcription factors [48]. The differences in the expression of these transcription factors may contribute to the diverse effects of ACVR1B activation in different cell types.

In skeletal muscle, ACVR1B mediates the myostatin signaling, whose dysregulation is associated with increased muscle mass in mammals [50,51,52]. Myostatin prefers ACVR1B in myoblasts, whereas it utilizes ALK5 in non-myogenic cells [53]. In previous studies, myostatin has been demonstrated to inhibit the myogenic differentiation of C2C12 cells [54,55]. ACVR1B inhibition promoted myogenetic differentiation in C2C12 in vitro, and reduced muscle mass and muscle fiber size in *ACVR1B*-silencing mice [18]. It is logical that miR-24-2 promotes differentiation by inhibiting *ACVR1B* expression to block myostatin signaling. However, some contradictory results have been reported in previous research studying the effect of myostatin on myogenic cell proliferation. Some studies have reported that myostatin inhibits cell proliferation [55,56,57,58]. Another study reported that myostatin stimulates C2C12 myoblast proliferation [59]. Such a contradiction may be a result of the alteration in gene expression caused by the immortalization process. Our study using cells freshly isolated from the fetal bovine skeletal muscle very likely revealed the actual function of *ACVR1B* in myogenic cell proliferation [60].

Other members of the miR-23a/27a/24-2 cluster also regulate muscle development. For example, miR-27a enhances myoblast differentiation, but the mechanism remains unclear [61]. Meanwhile, some studies have found that miRNA-23a/27a inhibits muscle atrophy [62,63]. The similar functions observed for miR-23a/27a/24-2 cluster members in myogenesis corroborate previous findings that miRNAs from the same cluster evolved to regulate functionally-related genes in a coordinated manner [64]. In this study, we demonstrate that bta-miR-24-3p accumulates and functions as a positive regulator during myogenic differentiation of fetal bovine PDGFRα^-^ progenitor cells.

## 5. Conclusions

Taken together, our findings reveal that bta-miR-24-3p promotes myogenic differentiation by directly targeting and suppressing *ACVR1B* expression. Therefore, our findings on bta-miR-24-3p and its target gene *ACVR1B* provide a mechanistic basis for related future research, and have great potential in improving bovine skeletal muscle differentiation.

## Figures and Tables

**Table 1:** Primers used for quantitative real-time PCR (qRT-PCR).

Name	Forward (5’–3’)	Reverse (5’–3’)
*MyoG*	CAAATCCACTCCCTGAAA	GCATAGGAAGAGATGAACA
*MYH1*	GGGAAACTGGCTTCTGCTGAT	TGGGTTGGTGGTGATTAGGAG
*MYH2*	GTCAAAGGGACTATCCAGAGCAG	AGAAGAGGCCCGAGTAGGTGT
*MYH4*	CTCCTAATCACCACCAACCCATA	TGTCAGCAACTTCAGTGCCATC
*MYH7*	AAGACAGTGACCGTGAAGGAGG	GGTTGATGGTGACGCAGAAGA
*ACVR1B*	TGCCCTCTGACCCTTCCATC	CACTCCCGCATCATCTTCCC
bta-miR-24-3p	CAGTGGCTCAGTTCAGCAGGA	
*18s*	GTAACCCGTTGAACCCCATT	CCATCCAATCGGTAGTAGCG
Cyclin D1	GACGAGCTGCTGCACATGGA	TGCTTGTTCTCCTCGGCCAC
Cyclin B1	TGGGAGAGACATAAACGG	TGGAAGCCAAGAGCAGTG
*PCNA*	GCTGTGTAGTAAAGATGCCT	ATCTCTATGGCAACAGCTTC
*CDK2*	TCTTTGCTGAGATGGTGACCC	TAACTCCTGGCCAAACCACC
*CDKN1A*	AAACGGCGGCAGACC	GCCCAAGGCAAAAGG

*MyoG*: myogenin; *MYH*: myosin heavy chain; *PCNA*: proliferating cell nuclear antigen; *CDK2*: cyclin-dependent kinase 2; *CDKN1A*: cyclin-dependent kinase inhibitor 1A.

**Table 2:** Primers used for vector construction.

Name	Forward (5’–3’)	Reverse (5’–3’)
Psi-CHECK-*ACVR1B*-W	ACTCTCGAGAACAACCACACACACAAAA	ATAGCGGCCGCGGATGAGAAGGTGCAACTT
Psi-CHECK-*ACVR1B*-Mut	TATCTGTCCTTGTACTGCTCCCCCCCGCCC	GGGCGGGGGGGAGCAGTACAAGGACAGATA
